# Mild-to-Moderate Gestational Iodine Deficiency Processing Disorder

**DOI:** 10.3390/nu11091974

**Published:** 2019-08-22

**Authors:** Ian Hay, Kristen L. Hynes, John R. Burgess

**Affiliations:** 1 Faculty of Education, University of Tasmania, Launceston 7250, Australia; 2 Menzies Institute for Medical Research, University of Tasmania, Hobart 7001, Australia; 3 Department of Endocrinology, Royal Hobart Hospital, Hobart 7001, Australia; 4 School of Medicine, University of Tasmania, Hobart 7000, Australia

**Keywords:** gestational iodine deficiency processing disorder, reading and language disorders, working memory capacity, speed of neuro transmitting ASD, ADHD, dyslexia, learning difficulties

## Abstract

This synopsis paper aims to identify if a common pattern of learning and social difficulties can be conceptualized across recent longitudinal studies investigating the influence of mild-to-moderate gestational iodine deficiency (GID) on offspring’s optimal cognitive and psycho-social development. The main studies investigated are: The Southampton Women’s Study (SWS)—United Kingdom; the Avon Longitudinal Study of Parents and Children (ALSPAC)—United Kingdom; the Gestational Iodine Cohort Longitudinal Study—Tasmania, Australia, and the Danish National Birth Cohort Case-Control Study—Denmark. In contrast to severe GID where there is a global negative impact on neurodevelopment, mild-to-moderate intrauterine iodine deficiency has subtler, but nonetheless important, permanent cognitive and psycho-social consequences on the offspring. This paper links the results from each study and maintains that mild-to-moderate GID is associated with a disorder that is characterized by speed of neural transmitting difficulties that are typically associated with working memory capacity difficulties and attention and response inhibition. The authors maintain that this disorder is better identified as Gestational Iodine Deficiency Processing Disorder (GIDPD), rather than, what to date has often been identified as ‘suboptimal development’. The Autistic Spectrum Disorder (ASD), Attention Deficit, Hyperactivity Disorder (ADHD), language and literacy disorders (learning disabilities and dyslexia) are the main manifestations associated with GIDPD. GIDPD is identified on IQ measures, but selectively and mainly on verbal reasoning IQ subtests, with individuals with GIDPD still operating within the ‘normal’ full-scale IQ range. Greater consideration needs to be given by public health professionals, policy makers and educators about the important and preventable consequences of GID. Specifically, more emphasis should be placed on adequate iodine intake in women prior to pregnancy, as well as during pregnancy and when lactating. Secondly, researchers and others need to further extend, refine and clarify whether GIDPD, as a nosological (medical classification) entity, is a valid disorder and concept for consideration.

## 1. Introduction

Iodine is a micronutrient that has been identified as being essential for optimal fetal neurodevelopment [1,2]. Iodine requirements increase for women during pregnancy because of the need for a rise in the production of maternal thyroid hormones, the transfer of iodine to the fetus, and increased renal clearance [3]. Iodine in the form of thyroid hormone is needed in all stages of the unborn child’s brain development, and so an adequate gestational iodine status is both important and necessary for normal fetal and new born brain development [4]. Maternal iodine deficiency (ID), predominantly in the first trimester of pregnancy, has been linked to long-term and persistent suboptimal development of the child, particularly with reference to cognitive, language and motor development [5,6,7]. Iodine deficiency (ID) is now recognized by the World Health Organization (WHO) as the most common preventable cause of brain damage [8,9,10] with more than 2 billion people from 130 countries at risk of neuro impairment from ID [11]. It is this preventable aspect that continues to motivate health professionals and policy makers across the world to work towards eliminating ID as an unnecessary risk factor to individuals achieving their full potential [9,10,12].

There have been several meta-analyses that have investigated the relationship between the impact of gestational iodine deficiency (GID) and children’s consequential achievement on intelligence quotient (IQ) measures. Bleichrodt and Born reported an IQ gap of 13.5 points between participants identified with GID, compared to their peers identified as non-iodine deficient [13]. More recent meta-analyses have also noted an IQ gap ranging from a 6.9 IQ points to a 10.2 IQ point reduction, depending on the level of fetal iodine deficiency (ID) for children under five years of age [14]. Qian and colleagues in their meta-analysis of Chinese studies also reported a reduction ranging from 4.8 IQ points to 12.5 IQ points depending on the children’s gestational iodine status [15].

Accepting that an IQ assessment is a valid procedure to investigating ID, there has, however, been an increased interest in research that has investigated ID that incorporates a wider range of assessment procedures [5,6]. In particular, in contrast to severe iodine deficiency where there is a clinically overt global negative impact on neurodevelopment [11], the impact of mild and mild-to-moderate GID is considered to have a subtler but identifiable manifestation on the offspring [6,7].

## 2. Selection of Studies

There have been a number of recent well developed and informed extended reviews that have summarized a large number of the previous GID findings [1,5]. Rather than replicate these mega GID reviews, the purpose of this paper is to use more of a synopsis and concept approach that has elements of phenomenological research [16,17,18] that is aiming to hypothesize the underlying mechanism, agency, or phenomenon that is likely to contribute to the production of the mild-to-moderate GID findings. In phenomenological/concept research, it is the analysis, understanding and hypothesizing of how a phenomenon manifests itself that is predominant.

The criteria for selection were the GID studies were recent, well conducted, longitudinal studies investigating mild-to-moderate GID, based on valid assessment and identification procedures of participants. Each study needed to represent a somewhat different approach to understanding GID to facilitate a more comprehensive insight into the condition. In this research approach, the analysis used a cognitive psychological framework to investigate GID, and was hypothesis driven, to identify if a common pattern of learning, social and behavior difficulties was distinguishable for individuals who were the offspring of mothers with mild-to-moderate GID.

Two of the studies selected were United Kingdom-based studies that utilized valid IQ instruments. The Southampton Women’s Study SWS [19] used the Wechsler Abbreviated Scale of Intelligence (WASI) [20] and the Avon Longitudinal Study of Parents and Children ALSPAC [21], the abbreviated form of the Weschler Intelligence Scale for Children III (WISC-III) [22] along with Neale Analysis of Reading Ability [23]. The third study is the authors’ Gestational Iodine Cohort (GIC) Tasmanian Longitudinal Study [24] that used the National Assessment Program—Literacy and Numeracy (NAPLAN) tests [25] and the Comprehensive Evaluation of Language Fundamentals (CELF-4) [26]. The fourth study examined was selected because of its difference to the other three GID studies, the Danish National Birth Cohort Case-Control Study [27] that reported on associations between maternal thyroid function and neurodevelopmental disorders as classified by the 10th revision of the International Classification of Disease (ICD-10) [28]. Each of these main studies is considered below. This is not to say that other similar studies were not worthy or are not discussed in this paper, but this is a concept paper and variation of design was a consideration in selection.

## 3. The Southampton Women’s Study (SWS)—United Kingdom

The Southampton Women’s Survey (SWS) study [19] (*n* = 654) identified that mothers’ pre-conception iodine status was linked to their children’s cognitive functioning at age 6 and 7 years. Although women’s pre-conception iodine levels are only an indicator of their gestational iodine levels, the relationship between the two is considered strong enough for the WHO [9] to recommend that all women planning to start a family increase their level of iodine in countries noted for ID. The Wechsler Abbreviated Scale of Intelligence was the cognitive measure (i.e., the Wechsler ‘vocabulary’ and ‘pattern matrix’ sub-tests). The study identified that ID was associated with a decrease in the children’s intellectual quotient (IQ) score, with a moderate pre-conception ID (<50 µg/g, iodine to creatinine ratio) associated with a mean 7.5 IQ point decrease (range 3 to12 IQ point reduction).

In addition to the Wechsler Abbreviated IQ scale, the SWS researchers also used three sub-tests from the Cambridge Neuropsychological Test Automated Battery [29,30] that were labelled by the test designers as ‘executive functioning’. As acknowledged by the researchers and the test designers, these three non-verbal, visuospatial sub-tests load on visual memory.

Unlike the IQ measure, the three Cambridge Neuropsychological visuospatial, non-verbal sub-tests labelled ‘executive functioning’ were not influenced by the mothers’ pre-gestational iodine status. This finding is somewhat counterintuitive because the Wechsler IQ verbal sub-tests are strongly associated with executive functioning [31,32,33]. For example, in the Weschler IQ vocabulary sub-test, the participant has to recognise the sound of the word and match this with a concept located in the participant’s long-term memory, then generate a reasoned response that explains the meaning of this word in context. As the IQ vocabulary sub-test progresses, the words become more abstract, for example: ‘What does the word democracy mean?’ This advancement in verbal reasoning and complexity requires the participant to utilize higher levels of proficiency in interpreting the question, and simultaneously start to rapidly retrieve separate bits of stored information from long-term memory, hold those items long enough to re-assemble the separate parts into a holistic response, that is then verbalised. Verbal IQ sub-tests load on working memory capacity, the ability to hold information long enough in memory to enable it to be connected into a schema, along with executive functioning, the ability to quickly reason and respond to the higher order cognitive task [31,32,33]. This contradiction in findings between the two different test results in the SWS study suggests that mild ID has a selective impact on cognitive functioning. The evidence is that sub-tests that have a heavier loading on verbal processing were more negatively affected by GID.

## 4. The Avon Longitudinal Study of Parents and Children (ALSPAC)—United Kingdom

The Avon Longitudinal Study of Parents and Children (ALSPAC) study demonstrated that adequate gestational iodine had a protective effect on the cognitive functioning of offspring based on results from the abbreviated form of the Weschler Intelligence Scale for Children III (WISC-III, UK) at age 8 and the Neale Analysis of Reading Ability at age 9 (*n* = 958 children). The WISC-III, measures verbal IQ (i.e., vocabulary and similarities) and non-verbal IQ (i.e., block design and matrix reasoning). The non-verbal IQ sub-tests load more on visual-spatial reasoning, and the verbal IQ sub-tests load more on verbal reasoning [34]. For example, in the verbal ‘similarities’ IQ sub-test, the participant is typically asked questions, such as: ‘What is the same about an apple and an orange?’ As this verbal IQ sub-test progresses the similarities become more abstract: ‘What is the same about anger and delight?’ This is also the case with the Weschler IQ sub-test ‘vocabulary’ with words becoming more abstract as the IQ sub-test progresses (e.g., What does the word ‘consider’ mean?).

The ALSPAC study reported significant difference in odds ratios between the adequate maternal iodine group (≥150 µg/g, iodine to creatinine ratio, *n* = 312) and the inadequate maternal iodine group (<150 µg/g, *n* = 646) for verbal IQ (*p* = 0.02), reading accuracy (*p* = 0.007) and reading comprehension (*p* = 0.02). Only the Weschler verbal IQ sub-tests (i.e., vocabulary and similarities) were significantly different between the two gestational iodine groups. Non-verbal Weschler IQ sub-tests that loaded on visual spatial reasoning were unaffected—a finding which aligns with the SWS study. Similar to the SWS study, the ALSPAC study lends support to the notion that mild-to-moderate GID has a selective negative impact on an individual’s cognitive functioning and not a global negative impact. This is in contrast to severe iodine deficiency where there is a global negative impact on neurodevelopment [11].

Examining the distribution of verbal IQ scores in the ALSPAC iodine study, the graphed information demonstrates that the adequate GID group was at the upper level of the average IQ range, mean around verbal IQ 112, with the scores clustering around this mean point. In contrast, the inadequate GID group were in the lower levels of the average range, mean around verbal IQ 105, with a greater spread of verbal IQ scores from this mean point. The ALSPAC and the SWS studies both used abbreviated versions of the Wechsler IQ Scale with both studies noting an approximate seven-point reduction in verbal IQ points.

This notion that mild-to-moderate GID selectively affects verbal IQ was also identified in a recent meta-analysis [35,36] based on data from three European pregnancy cohorts: Generation R in the Netherlands [37], the INfancia y Medio Ambiente Project (INMA) in Spain [38] and the already discussed ALSPAC study [21]. Summarizing this meta-analysis, it was reported that lower urinary iodine-to-creatinine ratios up to week 14 of gestation were associated with poorer verbal IQ scores (mean 6 IQ points difference across the three cohort studies), but there was no association of maternal iodine status with children’s non-verbal IQ scores.

In the ALSPAC study, the children in the inadequate gestational iodine group also demonstrated significantly lower performances on the Neale Analysis of Reading Ability tests for reading accuracy (reading the word correctly, *p* = 0.06) and reading comprehension (understanding what they read, *p* = 0.04). Compared to the inadequate iodine group, the adequate group had higher mean scores for word level accuracy and reading comprehension and greater clustering of the reading scores around the mean.

The ALSPAC study is particularly important from a psycho-cognitive perception because it has used a well-validated reading assessment instrument—the Neale Analysis of Reading Ability. Editions of this reading test have had a long history of research usage with students with reading difficulties [39,40] which in the UK is linked to the study of dyslexia [41] and in the USA is linked to the study of learning disability [34]. The ALSPAC study is helping to shift the research orientation for mild-to-moderate GID from just an intellectual impairment orientation, to the processing disorders of learning disability and dyslexia. Reading difficulties, as identified on tests such as the Neale Analysis of Reading Ability, are associated with three interconnected types of cognitive processes that are linked to working memory capacity and executive processing [42,43].

Typically, the first cognitive processing difficulty is that the reader has difficulties developing a core set of sight words that are quickly and automatically recalled and retrieved from the reader’s long-term memory [44]. The second typical cognitive processing difficulty is the reader’s word decoding skills and an inability to fully segment and blend the different letter combinations into their corresponding phonological units [43,44]. Thus, the reader typically guesses the word from either the orthographic pattern of the word or from those parts of the letters known to the child. This decoding difficulty is allied to the reader’s auditory and/or verbal working memory functioning [45,46] along with the reader’s inability to quickly synchronize and process phonological and orthographic information [46,47,48]. The third typical cognitive processing difficulty associated with a reading difficulty is language related. In this situation, the reader has a limited understanding and recognition of specific words or ideas in the text passage, so the reader either stops reading or guesses and/or substitutes a related word based on the general context of the story [44,49]. Connected to this language-related difficulty, the reader typically has difficulties with the syntax and/or semantics of the language used in the text, such that the child again either stops reading or guesses and/or substitutes a related word to the one in the text [50,51]. In terms of comprehension difficulties, if the reader has difficulties at the word and/or at the syntax and semantic language levels, the reader has difficulty in quickly forming a consistent schema (understanding) of what is read and so meaning from the text is reduced or lost [50,52,53].

Researchers using the Neale Analysis of Reading Ability in association with listening comprehension tests have also identified that children with poor reading comprehension (as noted in the ALSPAC iodine study) typically displayed poorer listening comprehension skills [54]. Poor listening comprehension is linked to students having a reduced ability to concentrate on and follow the teacher’s classroom instruction [39,47]—a cumulative risk factor for the student not fully benefiting from classroom instruction [34,41]. Poor listening and reading comprehension are linked to less fluent speed of processing [55,56], a reduction in working memory capacity [57,58,59], and a reduction in executive inhibitory control, that helps to maintain the student’s on task concentration [32,33,34].

The pattern of results in the ALSPAC study, where the low maternal iodine group had significantly reduced reading comprehension, word recognition and verbal IQ functioning, but not non-verbal IQ performance, is a typical pattern associated with children with a reading and/or a learning disability [41,60]. Such a finding in the ALSPAC study, connects GID with the traditional definition used in the USA for students with a learning disability [34]: Learning disabilities is a generic term that refers to a heterogeneous group of disorders manifested by significant difficulties in the acquisition and use of listening, speaking, reading, writing, reasoning or mathematical abilities. These disorders are intrinsic to the individual and presumed to be due to central nervous system dysfunction. Even though a learning disability may occur concomitantly with other handicapping conditions (e.g., sensory impairment, mental retardation, social and emotional disturbance) or environmental influences (e.g., cultural differences, insufficient/inappropriate instruction, psychogenic factors), it is not the direct result of those conditions or influences ([34], p. 11).

## 5. The Gestational Iodine Cohort Longitudinal Study—Tasmania, Australia

The third study examined is the authors’ Gestational Iodine Cohort (GIC) Tasmania Australia longitudinal study, comparing the effects of in utero iodine levels on children’s school achievement [61] and its follow-up study investigating the students’ school achievement and language performance [24]. Although the second published Tasmanian study did not use IQ as an outcome measure, it did use a standardized and psychometrically valid language assessment test, the Comprehensive Evaluation of Language Fundamentals 4th Edition (CELF-4). Similar to the ALSPAC Study, this study applied WHO cut-points used for determining iodine adequacy in a pregnancy population sample to individual women, where adequate maternal iodine during gestational was defined as a urinary iodine concentration at or above 150 µg/L and inadequate if below this benchmark [62]. The adequate and the inadequate gestational iodine mothers in this study were shown to be similar in terms of their socio-economic status.

A key outcome measure for both published Tasmanian studies was the students’ (*n* = 266) performance on national tests, (the National Assessment Program—Literacy and Numeracy, NAPLAN). The NAPLAN results on the same students were obtained for Years (Grades) 3, 5, 7, and 9. The NAPLAN scores are Rasch modelled so the different tests are comparable across time [63]. The key finding was that the students from the low maternal iodine group, across the year levels, demonstrated reduced performances on reading comprehension, with spelling measures the most affected by GID. As authors, we suggested that the type of NAPLAN spelling test used highlighted an underlying weakness with working memory capacity because the test employed homophone words embedded in a text passage (e.g., flower or flour; bare or bear, their or there) to assess the students’ spelling ability. This form of test increases the cognitive load on working memory capacity and reduces the processing speed, because the first word acts as interference in retrieval of the second word from long-term memory, with the reader also needing to rapidly and simultaneously use high levels of phonographical (auditory pathways) and orthographical (visual pathways) memory capacity in this executive functioning reasoning task [42,45,56,57].

The Tasmanian study [24] also followed up a subset of students (*n* = 34) using a psychometrically valid standardized measure of language functioning, the CELF-4. Although the CELF-4 does not purport to be a measure of verbal IQ, it does have many of the features of a verbal IQ test. Each of the CELF-4 index measures is made up of a composition of sub-tests and calibration to scaled index mean of 100. As noted in the 2017 Tasmanian paper [24], there was an approximate eight-point difference in performance based on sufficient versus deficient gestational iodine status for expressive language index. This finding aligns with the ALSPAC and the SWS studies where there was around a seven-point verbal IQ difference, between the iodine-sufficient and iodine-deficient groups.

Within the CELF-4, there is a working memory index, but this index is a composite score from short-term memory recall tasks. This is an important distinction to working memory capacity that requires the student to hold and quickly process a number of items in working memory and reason with this information into a higher order thinking and expression language output [39,40,41]. The CELF-4 ‘formulating sentences’ and ‘expressive language’ measures were both reduced for the inadequate gestation iodine group and each of these measures is linked to verbal working memory capacity. This is because these verbal tasks require the student to quickly locate, synchronize and process information from different stored cognitive locations and then use executive functioning to generate a logical verbal response to the question asked [52,54,56]. For example, in the ‘formulated sentence’ subtest task, the student is given a word and a picture and has to logically link the word to the different cues in the picture and generate a meaningful complex sentence that fits the narrative of the picture. This is a much more complex memory retrieval task, compared to just repeating back words or numbers. Again, as noted in the NAPLAN spelling tests (that used homophone words embedded within a text passage), the students in the low gestational iodine group demonstrated significantly poorer performance, compared to their adequate iodine peers. It is hypothesized that, compared to the adequate iodine group, the neurological networking of the inadequate iodine group becomes more ‘stressed’ and so less effective when more complex and extended verbal information is being processed and synchronized.

The Norwegian Mother and Child Cohort Study [64] also connects into this hypothesis, that neurological processing speed and working memory deficiency are linked to mild-to-moderate GID. The Norwegian study identified that suboptimal gestational iodine intake was associated with children at age three having higher levels of language delays and higher levels of anxiety. This association between language delays and mild-to-moderate GID has been replicated in a separate Norwegian study [65] using the psychometrically valid Bayley Scales of Infant and Toddler Development [66]. This study reported that suboptimal gestational iodine intake was strongly associated with lower infant expressive language skills (*p* = 0.002) and lower receptive language skills (*p* = 0.025) in infancy and toddlerhood, but not with reduced general cognitive or fine- and gross motor skills. Again, it is the selective impact of mild-to-moderate GID on higher order verbal neurological processing, associated with expressive language that was most affected.

Similar to the Tasmanian study, the Norwegian Mother Child cohort has also followed up the same cohort of individuals with different levels of gestational iodine. From a research perspective, this longitudinal analysis helps to clarify if GID produces a ‘trait’ that endures in some form across the life span, or GID is a ‘state’ that fades over time. To date, there have been two follow-up studies reported from the Norwegian Mother and Child Cohort Study. The first, identified that suboptimal gestational iodine intake was associated with children having higher Attention Deficit, Hyperactivity Disorder (ADHD) symptom scores at age 8 years [67]. The second follow-up study, reported that suboptimal gestational iodine intake was associated with poorer child language skills (*p* = 0.013), poorer reading skills (*p* = 0.019), and poorer writing skills (*p* = 0.004) as well as poorer school test result in reading (*p* < 0.001) and an increased likelihood of the child receiving special educational services (*p* = 0.042) at age 8 years [68].

The GIS Tasmanian study did not directly investigate developmental disorders, such as Autism Spectrum Disorder (ASD)—a developmental disorder that affects communication and behavior [69]. Even so, the poorer performance by the low gestational iodine group on the CELF-4 sub-test ‘formulating sentences’, does indirectly link this research finding to ASD. This is because researchers, who have compared students with high functioning Autism (IQ >80), to their non-ASD peers, identified that on the CELF-4 sub-test ‘formulating sentences’, the ASD students had significantly poorer performance [70]. This possible linkage between ASD and gestational iodine deficiency will be explored more in the following section.

## 6. The Danish National Birth Cohort Case-Control Study—Denmark

The fourth study is the Danish nationwide case-cohort study [27]. This study identified that abnormal maternal thyroid functioning in early pregnancy was associated with epilepsy, ASD, and the behavior disorder of ADHD (Attention Deficit, Hyperactivity Disorder) in the offspring. The study did, however, note that these associations differed by sub-types of exposure and by child age and sex. This notion that there is an association between maternal thyroid function, gestational iodine levels, ASD and ADHD is not an isolated claim.

With reference to ADHD a longitudinal study conducted in North-East Sicily compared two locations, one ID and one iodine adequate. It identified that ID was associated with higher rates of ADHD (68.7% difference by location) and lower IQ scores for children at 8–10 years of age, with a mean difference of 18 IQ points (ID location, IQ 92.1 ± 7.8; adequate iodine location, IQ 110 ± 10, *p* < 0.00005) [71]. This study is also relevant because it indirectly illustrates that adequate iodine before conception, during pregnancy and when the mothers are lactating, is a protective agent to neurodevelopment. Fetene et al., in their gestational thyroid review [72], concluded that low and high thyroid hormone levels and autoimmune thyroiditis during early pregnancy were associated with several offspring behavioral and psychiatric disorders, such as ADHD, ASD, pervasive developmental problems and externalizing behavior, in addition to epilepsy and seizure. The hypothesis that there is an association between gestational iodine deficiency and offspring developing ASD has also been identified and explored in previous investigations on this topic [73,74].

Connecting the Fetene et al. and the Danish cohort case-control findings, with the other findings explored in this paper, research into the ASD condition has identified that children with ASD have more difficulty with fast processing of verbal [75] and non-verbal visual and social information [76]. Non-verbal visual and social neuro-processing includes, ‘reading’ other people’s facial emotions and body language and being sensitive to the social context in which the person with ASD is located. For example, if a topic is of interest to the person with ASD there is a greater likelihood of monopolizing the conversation, and having difficulty accepting another person’s perspective on the topic [76,77]. In terms of these last two points, such rigidity and inflexible behavior and thinking is characteristic of executive functioning and processing difficulties with ASD [78,79,80]. That is, the neuro-pathways that help individuals to self-regulate and self-monitor their thinking and behaviors are considered to be less effective for individuals with ASD [81]. Executive function difficulties for individuals with ASD also involve an inability to suppress irrelevant or interfering information and impulses [79,82]. For example, in the classroom an inability for the child with ASD to concentrate on the task, if there is background noise or environmental movement and simulation, [83] and in the community for the person with ASD to become anxious within a noisy and crowded setting [77,80]. These executive function processing difficulties are also linked to working memory capacity difficulties with individuals with ASD having slower reaction time and a higher rate of perseveration when responding to questions [84]. Consequently, individuals with ASD are associated with difficulties in maintaining dialogue conversations and social interactions [76,77,78], and having higher levels of social anxiety and related anxiety disorders [85] including loneliness and depression [77]. It needs to be restated that the ASD disorder is a separate disorder to intellectual impairment and while there can be comorbidity between these conditions they are distinct and ASD cannot be diagnosed using an IQ assessment [70].

ADHD is also a distinct disorder, and it also aligns with the slower neurological functioning hypothesis, because it is the neurological network that controls impulse control and attention and response inhibition that is also slower [86,87] for children with ADHD. In addition, individuals with ADHD also have associated difficulties with processing information and working memory capacity [88,89]. Thus, ADHD, reading, spelling, learning and communication difficulties typically have high levels of comorbidity [90,91,92] because each of these difficulties is associated with processing speed, concentration, working memory capacity, and executive function difficulties [93,94]. This high level of connection is leading to the hypothesis that the disorders are more appropriately described as co-occurring, rather than comorbid [91,92]. Co-occurring implies that their underlying pathophysiologies are more causally related and so the disorders are not independent of each other, which is assumed when the disorders are comorbid.

## 7. Syntheses of the Studies

This paper joins a growing body of international research that is concerned about GID and its negative long-term consequences on the individual. Two factors are highlighted in this synopsis. The first, gestational iodine intake, is on a continuum and although severe iodine gestational deficiency is linked to more severe consequences, mild-to-moderate iodine gestational deficiency is also having a negative consequence on the child. The second factor highlighted is the association between intrauterine iodine deficiency and slower neural processing speed, which is linked to slower impulse control and response inhibition for individuals with ADHD and ASD and impaired working memory capacity. Consequentially, as noted in the studies explored, mild-to-moderate GID is linked to students with learning disorders, such as reading difficulties (i.e., dyslexia and learning disabilities), language delays, ASD, and ADHD, who have a disorder that is characterized by speed of neural processing and transmitting difficulties that are typically associated with working memory capacity difficulties and attention and response inhibition.

When GID is severe, there is a global negative impact on neurodevelopment and the severe form of intellectual impairment caused by ID and identified as cretinism. [95]. Approximately 5%–10% of pregnant women who are severe ID give birth to a child with cretinism [96]. In those world locations with endemic cretinism, there is also another cohort of children (5%–15%) who are identified as sub-cretins, who also have impaired intellectual functioning with an IQ of 50–69 [11]. Not to underestimate the importance of preventative iodine programs to eliminate cretinism and sub-cretinism, it may have indirectly had the effect of conceptualizing GID as a condition associated more at the lower end of the IQ distribution continuum. In contrast to severe iodine deficiency, mild-to-moderate intrauterine iodine deficiency has subtler, but nonetheless important permanent cognitive and psycho-social consequences on the offspring.

## 8. Gestational Iodine Deficiency Processing Disorder

Acknowledging the impact mild-to-moderate GID, as a disorder, has on offspring, the authors suggest that it could be better identified as Gestational Iodine Deficiency Processing Disorder (GIDPD) rather than, what to date has often been identified as ‘suboptimal development’. In this, we are suggesting the existence of a nosological (medical classification) entity involving specific aspects of neurocognitive development and GID, called GIDPD, which is implicated in specific learning disorders and probably Autism Spectrum Disorder. The rational for this nosological entity is that GID is already considered a spectrum disorder [1,7,12] that affects the neuro-networking [2,4,8] and, as already highlighted, mild-to-moderate GID is more associated with information processing difficulties. The term GIDPD helps to position GID research within a domain that is researching working memory capacity, attention and response inhibition. It also facilitates a shift in focus from conceptualizing intellectual status, as measured by IQ tests, as the dominant research paradigm in understanding mild-to-moderate GID. Whilst there is still value in this research paradigm, as noted in this paper, most individuals linked to mild-to-moderate GID typically have a reduction of some six to seven verbal IQ points and so are typically still within the normal full IQ range. Critically, these individuals are also likely to have some level of neuro-processing disorder that will impact on their overall learning and psycho-social development. The term GIDPD thus assists to co-locate GID within a broad and significant research public policy framework and highlights that GID is allied with a spectrum of disorders.

The focus on information neuro-processing also helps to balance the perception that GID is mainly associated with a low incidence per population severe intellectual impairment disorder, to a perception that GID is also associated with high incidence per population literacy, language, social and behavioral disorders. On this point, research on the prevalence rates of GID notes that the majority of women are in the mild-to-moderate iodine deficiency range. For example, in the Norwegian Mother and Child Cohort Study [62], there is a typical bell-shaped distribution of gestational iodine intake from food (in micrograms per day), with 74% of the 48,297 mothers sampled having an estimated intake from food lower than the Institute of Medicine Estimated Average Requirement during pregnancy of 160 μg/d [18] (3.7% of mothers sampled <50 μg/d; 29.3% of mothers 50 μg/d to 100 μg/d; 36.6% of mothers 100 μg/d to 150 μg/d). In a connected Norwegian Mother and Child Cohort study, urinary iodine concentration (UIC) was also measured in a subsample comprising 2910 pregnancies [97]. Of these, 37% had inadequate iodine (UIC below 50 µg/L). The median UIC was 59 µg/L in non-users of iodine containing supplements at the time of sampling (collected gestational weeks 17 to 20), and 98 µg/L in supplement users. Maternal iodine distribution patterns were also noted in the ALSPAC [21] and the SWS [19] studies.

Investigations on the prevalence rates of children with some level of developmental or learning disorder note that the majority of such children have a mild-to-moderate learning difficulty [98,99,100]. For example, in the USA the largest cohort of students with a special need are students with a learning disability (35%) who typically have a reading related disorder [99], followed by students with a language and speech impairment (20%) [98]. In Australia, the indications are that about 30% of children in the early years of schooling have some delay, typically related to their language and reading development [101]. In terms of ADHD, it is estimated that the worldwide-pooled prevalence of ADHD is 5.3% in the population up to 18 years of age [102]. The indications are that prevalence rates of ASD are on the increase, with the estimated prevalence of US children with a parent-reported ASD diagnosis now 1 in 40 (2.5%), with rates of ASD-specific treatment usage varying by children’s sociodemographic and co-occurring conditions [103]. The prevalence of serious intellectual disability (IQ <50 with deficits in adaptive behavior) in the United States and other developed countries is consistently identified to be in the range of 2.5 to 5 per 1000 children (0.25% to 0.5%) and that children in the mild-to-moderate intellectual disability range (IQ 50 to IQ 70) vary from as low as 2 to more than 30 per 1000 (0.25% to 3%) [104].

## 9. Triangulation

Across the four main studies examined in this paper, there is triangulation that adequate gestational iodine intake was a protective factor against the likelihood of the child having suboptimal development on one or more measures of cognition, reading, spelling, language, or behavior. Rather than treating each of these disorders in isolation, the evidence from this synopsis implies that clinicians and researchers need to consider the possible overlap of these conditions. GIDPD is hypothesized to be an underlying pathophysiology factor in understanding the aetiology and manifestation of each of these conditions. This is not to claim that other significant aetiological risk factors, along with genetic and DNA variants and home and social environmental factors are not also contributing to the development of these disorders. Even so, the findings, across different studies, countries, and testing procedures are converging on the GIDPD hypothesis that mild-to-moderate GID is an aetiological risk factor that affects the ability of the individual to quickly process information and so has an impact on working memory capacity and the individual’s performance on literacy, verbal processing, language, and social relationship measures.

This paper is not claiming that all individuals with a language and/or literacy and reading disorder or who have some level of ASD and ADHD have their aetiology with GID, but the studies reported in this paper lend support to the hypothesis that there is a level of comorbidity and co-occurrence between mild-to-moderate learning and behavioral disorders and mild-to-moderate GID. The logical proposition is that a mild GID is likely to correspond to a milder suboptimal neurodevelopment disorder. This milder disorder is, however, still detrimental to the individual’s ability to engage fast neuro-processing, which in turn affects the individual’s learning, social interactions and behaviors. Accordingly, a severe GID is likely to correspond to severe neuro-development-based disorders for the individual. This pattern reflects the maxim that the dosage and the levels of exposure affect the aetiology and level of severity of the manifested condition [105].

## 10. Public Awareness

Given that ID is far from a new health issue, the indications are that the health message about GID has a lower than warranted public health profile [106]. Although ID disorders are impacting on developing nations [3,7,8], mild-to-moderate ID, as evident in the studies reported in this paper, is also affecting mothers and their children in developed countries. Many expectant mothers are either unaware of the dangers of mild-to-moderate GID or unaware of what actions they need to take to maintain their iodine levels before, during and after pregnancy to try to prevent GIDPD [6,8,10]. The indications are that pregnant women in developed countries are more likely to have GID because of voluntary diet restrictions, either unknowingly or knowingly (such as, vegans and vegetarians) choosing to avoid or restrict their intake of foods high in iodine, such as dairy products, eggs and sea foods [107,108,109]. Certainly, women’s self-perceptions about their physical appearance and body image is strongly influenced by the popular media [110], which can place undue social pressure on women, planning to become pregnant, to inadvertently restrict their diet of micronutrients in an effort to maintain a specific body image [111,112,113].

In Australia, nutrition education is part of the national school curriculum subject called ‘Health and Physical Education’ [114]. Nutrition is repeatedly taught across the school years under the heading of ‘Healthy Eating’ and although topics such as obesity and a balanced diet are studied, the importance of micronutrients has little exposure. Unless the topic of ID in pregnancy is introduced at the upper end of high school, there is little likelihood that young women will be aware of the need and the steps required to maintain adequate iodine in their diet and prevent GIDPD, associated with mild-to-moderate ID. It is this need to raise the public profile of GID and its preventable nature that is important for all young people to understand. Although the medical profession has a significant role in educating all women of fertility age about ID and pregnancy, this education role needs to be a shared role, so it involves nutritionists, health professionals, educators, the media, and governments.

## 11. Implications and Research Direction

In addition to greater public educational programs on maternal ID, from a research and reporting perspective there are at least two considerations. The first issue is the need for a variety of assessment measures to be used when investigating how different levels of gestational iodine exposure manifest across the life span. This is because a disorder manifests itself differently over time and often demonstrates a high level of comorbidity with secondary impairments linked more to psychological wellness [99,105]. For example, in the early school years, the concern with the individual may be more associated with reading or spelling difficulties, but in adolescence and adulthood the concern may manifest itself more around the individual’s psychological wellness status [115,116]. The recognition that GIDPD is likely to have unexpected long-term negative consequences is supported by the longitudinal research into individuals with learning difficulties and dyslexia. The evidence is that individuals with learning and reading difficulties as youths and as adults have higher rates of prison and juvenile detention [117]; homelessness [118]; substance abuse [119]; emotional difficulties [120]; and suicide [121]. Secondly, the indications are that GIDPD affects the individual across the life span [19,21,24,27], but how to manage, reduce or eliminate this disorder’s negative consequences on the individual, across the life span, still needs more research attention.

If it becomes more recognized in the public education and psychology domains that there is a possible neurological processing basis for performance differentiation across children, there are implications. The first is a recognition that the child with a reading and/or spelling difficulty is not ‘lazy’ and if he/she just worked harder the difficulty would ‘disappear’ [99,122]. The second implication is to make more accommodations for students with weaker working memory capacity [31,83,123]. Such accommodations include an increase in wait time when questioning students and reducing the level of verbal load the student is required to process at any one time, by breaking up the instruction into shorter sections and including more visual learning. Assessment tasks, particularly in high schools and universities, that are timed can be a problem to students with weaker working memory capacity [122]. Thus, providing more time in examinations and with tests is an effective strategy. For example, when adults with dyslexia-related reading problems were given an untimed reading assessment task, these readers performed at a similar level to their peers who did not have dyslexia-related reading problems [124].

IQ and other standardized tests are logical tools to investigate GIDPD across the life-span. Even so, just reporting the total IQ full score reduces the ability to conduct a profile analysis across the different IQ sub-tests designed to investigate different aspects of cognition. Importantly, focusing more on different aspects of cognition assists in gaining a greater understanding of the often subtle pattern of differentiation between cohorts, and increases the likelihood that triangulation across different research approaches and studies can be achieved. For example, the hypothesis that neuro-processing and so working memory is affected by GIDPD triangulates well with rodent studies that have demonstrated that GID is associated with delayed axonal growth in the hippocampus [125] and a decrease in myelin basic protein (MBP) [126] that influences myelination. Importantly, thyroid hormones, which are synthesized from iodine, promote the myelination process during in utero development [4]. Myelin insulates nerve axons to increase the speed at which information, encoded as an electrical signal, transfer from one nerve cell body to another in the central nervous system [127]. The evidence is that the hippocampus plays a critical role in memory formation and in memory recall and recognition by providing the brain with a spatiotemporal framework within which the various sensory, emotional, and cognitive information components of an experience are connected together [128,129]. This framework allows the experience to be stored in such a way that it can be later effectively and quickly recovered and retrieved from long-term memory and so processed as a conscious recollection of that experience [130]. Thus, impairment of the hippocampus and impairment of myelination would both have an effect on the speed in which memories are retrieved and activated and the speed of neuro-transmitting. Although rodent studies are not human studies, such findings have relevance to the hypothesis that neuro-processing speed of information at the axon level is affected by GIDPD, resulting in the individual having reduced working memory capacity and attention and response inhibition. GID in its different forms can be considered as a set of related disorders, but its comorbidity and co-occurrence with other learning, social and behavior disorders also need to be highlighted and this is facilitated by the conceptualization and the identification of GIDPD.

## 12. RCT versus Observational Studies

In the world of evidence-based pharmaceutical medicine, the associations summarized in this paper are all from observational studies and so a lack of randomized control trial (RCT) studies may apparently ‘devalue’ these studies. The issue of RCT versus observational studies in medical and human development research is not a new concern, but the notion that RCT is superior to observational studies has been challenged [131,132]. In terms of public health research, the claim is that observational studies can provide superior evidence to that possible from an RCT research design [133]. Even so, RCT and the observational studies reported in this paper are not as dichotomous as sometimes suggested as both are investigating a possible causal relationship between variables. In the observational studies reported, the independent variable is the level of gestational iodine and the dependent variable is the forming fetus’ neurodevelopment. The confounding variables are the other variables, apart from the level of gestational iodine, that may also impact on the fetus’ neurodevelopment. Both forms of research aim to identify two comparison cohorts, that differ in gestational iodine, but are similar on confounding variables and so ‘controlled’ for in the research design. Examples of confounding variables are socio-economic variables of mothers, health and age variable of mothers, location variables, and quality of medical, health, and other care variables of mothers and fetus.

The term observation may suggest a lack of rigor in the assessment, but the mothers’ gestational iodine levels were calibrated using standardized biochemical pathology procedures to international standards, and the children’s cognitive and related assessments were based on objective psychometrically strong assessments, such as the Weschler IQ tests and the CELF tests. RCT and observational research should be seen as being complementary, rather than in competition. For example, the rodent RCT studies [4,125,126] have provided triangulation to the observational studies. The longitudinal observational research studies reported in this paper have identified that the impact of GID is demonstrated years away from the first trimester of the fetus’ and so the child’s life. This context lends itself to an observational study.

Much of the debate around the consequences of suboptimal iodine intake is the lack of randomized control trial (RCT) studies documenting the beneficial effects of iodine supplementation to pregnant women [96,134]. Ethically, it is virtually impossible to conduct an RCT with iodine supplementation versus placebo in mild-to-moderately iodine-deficient women, because of the high and known risk from observational studies of ID and neuro-disorders and the findings from the cretinism research [1,2,3,4,5]. In Australia, medical human research standards require participants to give informed consent, with a stress on understanding the consequences of participating in medical research. Even if the mother agreed to participate in a placebo trial, the consequences of harm to the fetus (child) would still rest with the researcher [135]. Controlling the confounding variables is just as difficult in both RCT and in observational research. For example, one of the main recent RCT studies that attempted to identify the beneficial effects of iodine supplementation to pregnant women [136] found no benefit of the intervention, but the study was invalidated because the pregnant women who participated turned out not to be iodine deficient [137].

There have been two RCT studies investigating cretinism in areas noted for ID that support iodine supplementation. The first conducted in the 1960s in Papua New Guinea used iodized oil and identified a reduction in the level of babies born with cretinism [138]. Importantly, the researchers concluded that iodized oil was effective in the prevention of endemic cretinism and that it should be given prior to conception. The second RCT study conducted in 1969 in Peru and Ecuador focused on a reduction in goiter severity [139]. Two years after the start of the research, the intervention group had no babies born with cretinism, but the non-intervention group was associated with babies born with cretinism. This again suggests the benefits of adequate iodine prior to conception.

## 13. Advice to Women and Iodine

The World Health Organization as well as health authorities in many countries recommend that pregnant women use iodine supplements to secure an increased intake during pregnancy [9,140], however, there is an increased recommendation to enhance the iodine status of women before they become pregnant [10,106,141]. The main evidence for this recommendation is the time lag to fully synthesize iodine into thyroid hormones, for although iodine is rapidly absorbed after ingestion, its synthesis and incorporation into the thyroid hormones and then into thyroid stores takes several weeks [142]. Initiation of iodine supplement use after conception may be too late to counteract the adverse effects that specifically occur in the first trimester, that are associated with long-term inadequate iodine intake [68]. This, along with the reality that many pregnancies are unplanned, many women are unaware that they are pregnant in the early stages and/or are unaware of the recommendations on supplementation preconception [143] has provided support for mandatory fortification of stable foods in countries with ID [96]. Thus, adequate iodine before conception, shares some similarities to adequate folic acid before conception, which also takes time to metabolize and has a ‘protective’ role in the first trimester [144]. It is this timing issue that, in part, helps to explain the lack of the evidence of any benefit of iodine supplement initiation in pregnancy [64,65,68]. On the contrary, just supplementation in the first trimester may result in a temporary ‘thyroid stunning’ (that is, temporarily inhibiting thyroid hormone production and/or release) [97,141], resulting in a possible adverse impact on the developing fetus [5,10]. Therefore, more emphasis must be placed on adequate iodine intake in women prior to pregnancy. In areas low in iodine, the health authorities respond to the evidence of adverse neurocognitive effects of mild-to-moderate iodine deficiency by advocating iodine supplements to pregnant women, while the group that should also be targeted to protect fetuses, are women of reproductive age. For women who live in low iodine areas, iodine supplementation is needed at the preconception, the pregnancy and the lactating stages. This is because an adequate thyroid hormone is needed across all phases of the unborn child’s and the new born child’s neurodevelopment, but especially in the first trimester [4].

## 14. Conclusions

Risk factors and protective factors are fundamental in understanding human development because an individual’s development is essentially cumulative in nature [145]. Logically, the more risk factors the individual has, the greater the likelihood of a detrimental impact on that individual’s potential on a range of wellbeing, economic, social, psychological and cognitive measures [105]. Thus, GID in its different forms has both an economic and a social cost to the individual as well as to society as a whole [5,8,12]. Critically, although there is still more to know, one of the major challenges now is to transfer what is already known into more effective public and school-based education and intervention programs.

This paper has described, through the four review analyses, the effects of mild-to-moderate iodine deficiency (ID) through diversified investigative tools that suggest the existence of a nosological (medical classification) entity involving specific aspects of neurocognitive development, called Gestational Iodine Deficiency Processing Disorder. GIDPD is implicated in specific learning disorders and probably Autism Spectrum Disorder (ASD). This has far greater implications than suggested by the reduction of some IQ points alone.

Therefore, the following statement summarises the core issues associated with the impact of mild-to-moderate Gestational Iodine Deficiency (GID): Iodine is a micronutrient and ID during pregnancy is linked to impaired central nervous system development in the first trimester, with ID identified as a preventable cause of neurological impairment. ID is directly linked to dietary intake and at the severe end of the continuum it causes cretinism, a severe form of intellectual impairment. At the milder end of the continuum, GID is associated more with reduced speed of neuro-transmitting and therefore reduced working memory capacity, attention and response inhibition, resulting in what may be termed Gestational Iodine Deficiency Processing Disorder. Although GIDPD can be studied as an independent condition, there is evidence of its comorbidity and co-occurrence with other developmental disorders, such as, Autistic Spectrum Disorder (ASD), Attention Deficit, Hyperactivity Disorder (ADHD), along with language and reading disorders (learning disabilities and dyslexia) and reduced performance on verbal IQ subtests.

Critically, the authors of this paper support the recommendation that in ID areas, women considering conception should be advised to take iodine supplementation for several months prior to pregnancy as well during and after pregnancy. The researchers also ask others to further extend, refine and clarify if the concept of GIDPD is a valid disorder and concept for consideration.
